# National Insurance and the Local Government Board

**Published:** 1917-09-29

**Authors:** 


### NATIONAL HEALTH INSURANCE ACT DEVELOPMENTS.

### National Insurance and the Local Government Board.

When Lord Rhondda accepted the position of Food Controller he allowed a statement to be made to the effect that he had only undertaken the arduous duties of that appointment on condition that the work upon which he had been engaged at the Local Government Board—namely, the institution of a Ministry of Health—should not suffer thereby. It was generally understood at that time that the plans for setting up the new Ministry of Health were well matured, and from the fact that Mr. Hayes Fisher, who had been prominently associated with Lord Rhondda in the advocacy of the new Ministry, was appointed as his successor in the office of President of the Local Government Board, it was anticipated that the introduction of the Bill to give effect to the proposed scheme would follow almost immediately. Some weeks have now passed and nothing has yet been done. Various excuses have been offered, such, for instance, as the abnormal congestion of legislative business in Parliament, but they have not really been convincing. As evidence of the hollowness of the excuse of Parliamentary congestion it may be pointed out that the "very short Bill" dealing with maternity and child-welfare, which Lord Rhondda had prepared, and which was merely designed to empower Local Authorities in England and Wales to exercise powers already possessed by similar authorities in Scotland and Ireland, has not been laid upon the Table of the House.

### The Real Reasons for the Delay.

It now appears that Lord Rhondda has met with opposition, and that the schemes have been held up in consequence. Although no longer officially connected with the Local Government Board, he presided at a meeting of the National Baby Week Council on July 30, and in the course of his remarks, as reported in the *Times*, he said that "the only opposition he experienced while he was endeavouring to secure the establishment of a Ministry of Health was from those who claimed to represent the approved societies."

With regard to the "very short Bill," which he had considered as an ordinary war measure, he is also reported to have said that one would have thought it inconceivable that such a Bill could be opposed for a single moment; but no sooner had he proposed it than Departments said, "You are infringing on our prerogative and trespassing on some of the ground that we intend taking up later." He had been informed that by such a Bill as he proposed it was possible to save the lives of a thousand babies a week, and yet that Bill had been delayed by the jealousy of different departments.

Here, then, apparently we have from Lord Rhondda's own lips the real reasons for the delay. It is no longer Parliamentary congestion, or the preoccupation of the Government on more urgent war measures, or the absence on war-service of many who would be affected by the proposed changes, which has to be blamed. But opposition of "vested interests" and departmental jealousies have stepped in, with the result that reforms which may be and have been characterised as urgent have been hindered.

### Is the Opposition Justified?

In pre-war days it is probable that the Bills would have been introduced into Parliament, and that when their terms became definitely known to the public, they would have formed the basis for free and open discussion and would ultimately have been modified according to the strength of the support which those who desired their amendment were able to secure. In these days of altered Parliamentary procedure, when it is only possible for the Government to allow uncontentious measures to be introduced, the free and open discussion in the House is replaced by the more secret and underground methods alone available. It is by no means certain that even under war conditions a reversion to the practice of unfettered Parliamentary discussions in matters of purely domestic politics would not be an advantage. As matters stand, one would be tempted to observe that the delay in the establishment of a properly organised Ministry of Health and in the introduction of reforms by which the lives of thousands of children are to be saved, is highly discreditable to those who have raised opposition to the schemes. The considerations above mentioned must, however, be borne in mind before any such hasty judgment is passed upon the attitude of National Insurance workers, or upon the representatives of Government Departments.

It does not follow as a matter of course that the plan proposed by a capable and energetic administrator like Lord Rhondda is necessarily the best that could be devised. It would even seem from his own words that he had not himself realised the importance of the work until he was appointed to the Local Government Board. How, then, was it possible within so short a period as six months for him to become so fully acquainted with the complicated mechanism of our present health administration that he could dare to suppose that any scheme of co-ordination, based upon recommendations chiefly from permanent officials of his own Department, would obtain the immediate and unqualified acceptance and approval of all parties?

### Objections not Fundamental.

Acceptance of the general principle of the desirability of setting up a Ministry of Health to coordinate the services now controlled by the Local Government Board, the Home Office, the Board of Education, the National Insurance Commission, the War Office, the Admiralty, and the India Office —to mention some only of the various authorities at present regulating certain sectional health administrative matters—does not necessarily imply

NATIONAL INSURANCE—(*continued*).

agreement with all the details in the proposed scheme of co-ordination. The opposition to which Lord Rhondda refers is not opposition to the general desirability of the establishment of the Ministry of Health. On the countrary, all sections of National Insurance workers stand to gain by the establishment of such a Ministry, provided that the work they are doing is recognised and given scope for development, and each section has now made it clear that no opposition is raised to the fundamental principles of such a Ministry.

The National Associations of Insurance Committees took the earliest possible opportunity of sending a deputation to Lord Rhondda to assure him of their support and to ascertain more fully his intentions. The Association of Approved Societies has now embodied the views of the approved societies in resolutions which have been sent to the Local Government Board as follows:—

"That, in view of Lord Rhondda's resignation of the Presidency of the Local Government Board and the reported postponement of the establishment of a Ministry of Health, this Association, as a matter of great urgency and of the utmost importance, places on record its opinion: (1) That it is desirable in the interests of the health of the community, uninsured persons as well as insured, for immediate steps to be taken to establish a Ministry of Health which will take over and co-ordinate the following health services" (then follows a list which need not be reproduced in full). As regards National Health Insurance, the resolution proceeds, "That the functions of the National Insurance Commission should form an integral part of such Ministry, and that approved societies should continue to administer the sickness, disablement, and maternity benefits under the National Insurance Act. Such societies, however, should not be burdened with additional expenditure necessitated by the active campaign of the Ministry of Health to seek out and stamp out disease wherever possible."

(2) "That such Ministry of Health should be independent of any existing Government Department, and under its own Minister responsible directly to Parliament."

### An Independent Ministry Indispensable.

It will thus be observed that the opposition of the approved societies, if it may be so called, is opposition to the concentration of the health administration under the ægis of the Local Government Board. The association of the Local Government Board with the administration of the Poor Law would impart to such Ministry, if based upon the Board, a taint of pauperism which it is held insured persons would resent. Moreover, the activities of the Board are conducted through Local Authorities, many of whom would be reluctant, judging by past experience, to take any steps for the betterment of health conditions for fear of increasing the burden of the rates.

It is claimed that the Insurance Committees are better adapted in constitution as the basis for the development of a forward policy in health administration. The committees must not be judged by the fact that no great advance has as yet been made through their agency. They have only been in existence for just over five years, they have been hampered all along by inadequacy of funds—even so far as the administration of sanatorium benefit, which was directly placed within their province—and they have been too frequently disregarded by the Local Authorities.

At the same time it has to be recognised that a forward and progressive health administration must immediately require the expenditure of large sums of money on housing, and as such expenditure would necessarily fall under the control of the Local Government Board it is imperative that the Board must be largely represented upon the new Authority.

### Jealousies Must Give Way to Conciliation.

In any scheme of co-operation there must be give and take. This principle of conciliation will not spring into existence of its own accord merely through the establishment of a new Ministry. It must commence beforehand. The publication of reports calculated to suggest that approved societies are working for some ulterior ends of their own is, therefore, to be deprecated. Dr. Saleeby, in the *Daily News and Leader* has even gone so far as to state that the colossal new vested interest of National Insurance as represented by the approved societies is "an interest not in health, but in disease." He refers particularly to the industrial assurance approved societies, and states that they "profit mightily by disease." How this can be he does not inform us. It is, in fact, difficult to see how anything which would tend to increase the amount of payments to be made by such societies or by the companies they represent could be any advantage to them. This is, however, by the way.

The point we desire to make is that free and open discussion with a view to obtaining the best possible health administration is not facilitated, but is rather hindered by imputing unworthy motives to those who may not hold precisely the same views as oneself on matters of method and detail.

